# Machine Learning‐Assisted Prediction and Generation of Antimicrobial Peptides

**DOI:** 10.1002/smsc.202400579

**Published:** 2025-03-06

**Authors:** Sukhvir Kaur Bhangu, Nicholas Welch, Morgan Lewis, Fanyi Li, Brint Gardner, Helmut Thissen, Wioleta Kowalczyk

**Affiliations:** ^1^ CSIRO Manufacturing Research Way Clayton Victoria 3168 Australia; ^2^ CSIRO Information Management & Technology Kensington Western Australia 6151 Australia; ^3^ CSIRO Information Management & Technology Research Way Clayton Victoria 3168 Australia

**Keywords:** antimicrobial peptides, antimicrobial resistances, bacteria, machine learning, multidrug resistances

## Abstract

Antimicrobial peptides (AMPs) offer a highly potent alternative solution due to their broad‐spectrum activity and minimum resistance development against the rapidly evolving antibiotic‐resistant pathogens. Herein, to accelerate the discovery process of new AMPs, a predictive and generative algorithm is build, which constructs new peptide sequences, scores their antimicrobial activity using a machine learning (ML) model, identifies amino acid motifs, and assembles high‐ranking motifs into new peptide sequences. The eXtreme Gradient Boosting model achieves an accuracy of ≈87% in distinguishing between AMPs and non‐AMPs. The generated peptide sequences are experimentally validated against the bacterial pathogens, and an accuracy of ≈60% is achieved. To refine the algorithm, the physicochemical features are analyzed, particularly charge and hydrophobicity of experimentally validated peptides. The peptides with specific range of charge and hydrophobicity are then removed, which lead to a substantial increase in an experimental accuracy, from ≈60% to ≈80%. Furthermore, generated peptides are active against different fungal strains with minimal off‐target toxicity. In summary, in silico predictive and generative models for functional motif and AMP discovery are powerful tools for engineering highly effective AMPs to combat multidrug resistant pathogens.

## Graphical abstract

A machine learning‐based in silico predictive and generative model is developed to engineer highly effective antimicrobial peptides (AMPs) for combating multidrug‐resistant pathogens. An iterative algorithm involving peptide generation, scoring, and motif identification produces AMPs with high theoretical scores. Experimental validation against microbial pathogens and algorithm improvement based on these results demonstrates 80% accuracy showcasing its effectiveness and translational potential.

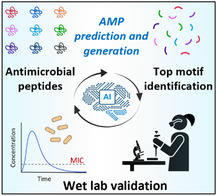

## 1 Introduction

The battle against infectious diseases and antibiotic‐resistant pathogens continues to evolve, and in this context, the development of novel antimicrobial agents is of paramount importance.^[^ ^1^ ^]^ The over‐ and misuse of conventional antibiotics have led to intensive increase in morbidity and mortality rates globally, due to high rise in multidrug‐resistant (MDR) infections.^[^ ^2^ ^]^ Antimicrobial peptides (AMPs) have emerged as promising candidates in this endeavor, owing to their potent, broad‐spectrum activity against a wide range of microorganisms.^[^ ^3^ ^]^ The clinical use of AMPs is still restricted to topical applications, for instance, in the treatment of wound and skin‐related infections due to their enzymatic degradation and quick kidney clearance. This drawback can be tackled through various delivery strategies/vehicles (like liposomes, metal nanoparticles, polymeric nanoparticles, lipid nanoparticles, etc.).^[^ ^4^ ^]^ Unlike conventional antibiotics that heavily rely on the interaction with specific target molecules, usually proteins, AMPs have a nonspecific mechanism of action. They often induce cell death by disrupting the plasma membrane and hence have low chances of antimicrobial resistance (AMR) development.^[^ ^5^ ^]^ However, the experimental discovery and optimization of AMPs can be a time‐consuming and costly process.^[^ ^6^ ^]^ Designing new peptides involves optimization of peptide sequences through truncation, alanine scans, etc., leading to the generation of large peptide libraries which are required to be validated through various biological studies. Therefore, in silico predictive models have emerged as indispensable tools for accelerating the discovery and design of effective AMPs. These computational approaches leverage the power of bioinformatics, machine learning (ML), and molecular modeling to predict the antimicrobial activity of peptides, thus guiding researchers toward the development of novel therapeutic agents.^[^ ^7^ ^]^ Here, we have explored in silico predictive models for AMP activity, examining their principles, methodologies, and potential impact on the field of antimicrobial research.

There are several databases dedicated to AMPs that provide valuable information about their sequences, structures, activities, and other related data. These include APD2 and APD3 which are comprehensive and widely used databases for AMPs.^[^ ^8^ ^]^ They offer a vast collection of experimentally verified AMPs, along with their sequences, structures, activities, and other pertinent information. Amino acid Physico‐chemical properties Database is an extension of APD3 and focuses on the structural aspects of AMPs, offering detailed information about their 3D structures and physicochemical properties.^[^ ^9^ ^]^ Database of Lantibiotics and Microcins is specialized in lantibiotics and microcins, which are ribosomally synthesized AMPs. It provides information on their structures, genes, and antimicrobial activities.^[^ ^10^ ^]^ Database of Antimicrobial Activity and Structure of Peptides provides a curated collection of AMPs, including information on their sequences, structures, and antimicrobial activities.^[^ ^11^ ^]^ It also offers tools for analyzing and predicting peptide properties. Collection of Anti‐Microbial Peptides (CAMP)^[^ ^12^ ^]^ and its extended version CAMPR3^[^ ^13^ ^]^ are databases that focus on naturally occurring AMPs. They also offer information on their sequences, structures, and antimicrobial activities. Users can input peptide sequences to predict their antimicrobial activity, and it ranks peptides according to their potential antimicrobial efficacy.^[^ ^13^ ^]^ These databases and webservers offer valuable resources that can assist in the discovery, design, and study of AMPs. These tools often employ ML algorithms and bioinformatics approaches to make predictions based on peptide sequences or structural features. Some common algorithms and methods used for predicting AMPs include support vector machines (SVM), random forest (RF), deep learning, k‐mer‐based, cross‐validation, evaluation metrics, etc.^[^ ^14^ ^]^ A supervised learning algorithm, SVM, is commonly used for AMPs prediction. It finds a hyperplane that can best separate AMPs from non‐AMPs in a feature space. Various features can include amino acid composition, physicochemical properties, and structural information.^[^ ^15^ ^]^ Another common ML tool is RF which is an ensemble learning method that combines the outputs of multiple decision trees.^[^ ^16^ ^]^ It can be used for AMP prediction by considering various sequence‐derived features and generating a consensus prediction. Deep learning techniques, such as convolutional neural networks (CNNs) and recurrent neural networks, have also been utilized for AMP prediction.^[^ ^17^ ^]^ These models can learn complex patterns and representations from peptide sequences. When datasets consist of large number of features, techniques like principal component analysis (PCA) or feature selection methods (e.g., chi‐squared feature selection) are used to reduce the dimensionality of input data and to select the most informative features for prediction.^[^ ^18^ ^]^ Moreover, cross‐validation and evaluation metrics are essentially used to assess the performance of prediction models to ensure their reliability.^[^ ^19^ ^]^

The choice of algorithm depends on various factors such as the dataset, the available features, and the specific research goals.^[^ ^20^ ^]^ Additionally, the continuous advancement of ML and bioinformatics techniques continues to enhance the accuracy and reliability of AMP prediction models. Features derived from the physicochemical characteristics which impact the interaction of peptides with bacterial membranes (hydrophobicity, amphiphilicity, charge density, propensity to the aggregation, etc.) can be used to provide inputs for ML models to predict AMPs.^[^ ^6^, ^18^, ^21^ ^]^ The structure‐based methods that consider the 3D structure of peptides, such as molecular dynamics simulations or docking studies, can also provide insights into AMP activity.[^21^, ^22^] Hybrid approaches (combination of models) can enhance the predictive power of models in designing and predicting AMPs.^[^ ^23^ ^]^

Herein, we have used physiochemical properties of known AMPs and non‐AMPs to build a predictive ML model. In addition, a generative model was built using ML‐based scoring of amino acids motifs from known AMPs and rearranging them to generate new peptide sequences of specified length for *n* number of generations to reach a high antimicrobial activity score. Identifying conserved motifs or patterns in AMP sequences can help to generate new AMPs. These motif‐based methods search for recurring sequence patterns associated with antimicrobial activity.^[^ ^23^ ^]^ This strategy is beneficial as the AMPs contain symmetrical sequence motifs or repetitive amino acid patterns which are imperative for their function and activity.^[^ ^24^ ^]^ The model was validated experimentally, where some of the highest ranked peptides generated using the algorithm were tested against bacterial pathogen: *Enterococcus faecium* ATCC19433, *Staphylococcus aureus* ATCC25923, *Klebsiella pneumoniae* ATCC43816, *Acinetobacter baumannii* AB5075, *Pseudomonas aeruginosa* PAO1, and *Enterobacter* ATCC13048. These pathogens are collectively referred as “ESKAPE” pathogens as they have the ability to escape the effects of antibiotics through various mechanisms, including antibiotic resistance genes, efflux pumps, biofilm formation, and the development of antibiotic‐tolerant persister cells.^[^ ^25^ ^]^ As a result, infections caused by ESKAPE pathogens are often difficult to treat and can lead to serious health complications and death. The study demonstrates the potential of algorithm in predicting and generating promising antimicrobial motifs and peptide candidates to tackle MDR microbial pathogens.

## 2 Results and Discussions

### 2.1 ML‐Based Prediction as AMP or Non‐AMP

The need for new ML prediction models for distinguishing between AMPs and non‐AMPs is driven by the urgent demand for novel antimicrobial agents, the challenges posed by antibiotic resistance, and the potential for computational methods to accelerate the discovery and development of effective antimicrobial therapies.^[^ ^26^ ^]^

**Figure 1:**
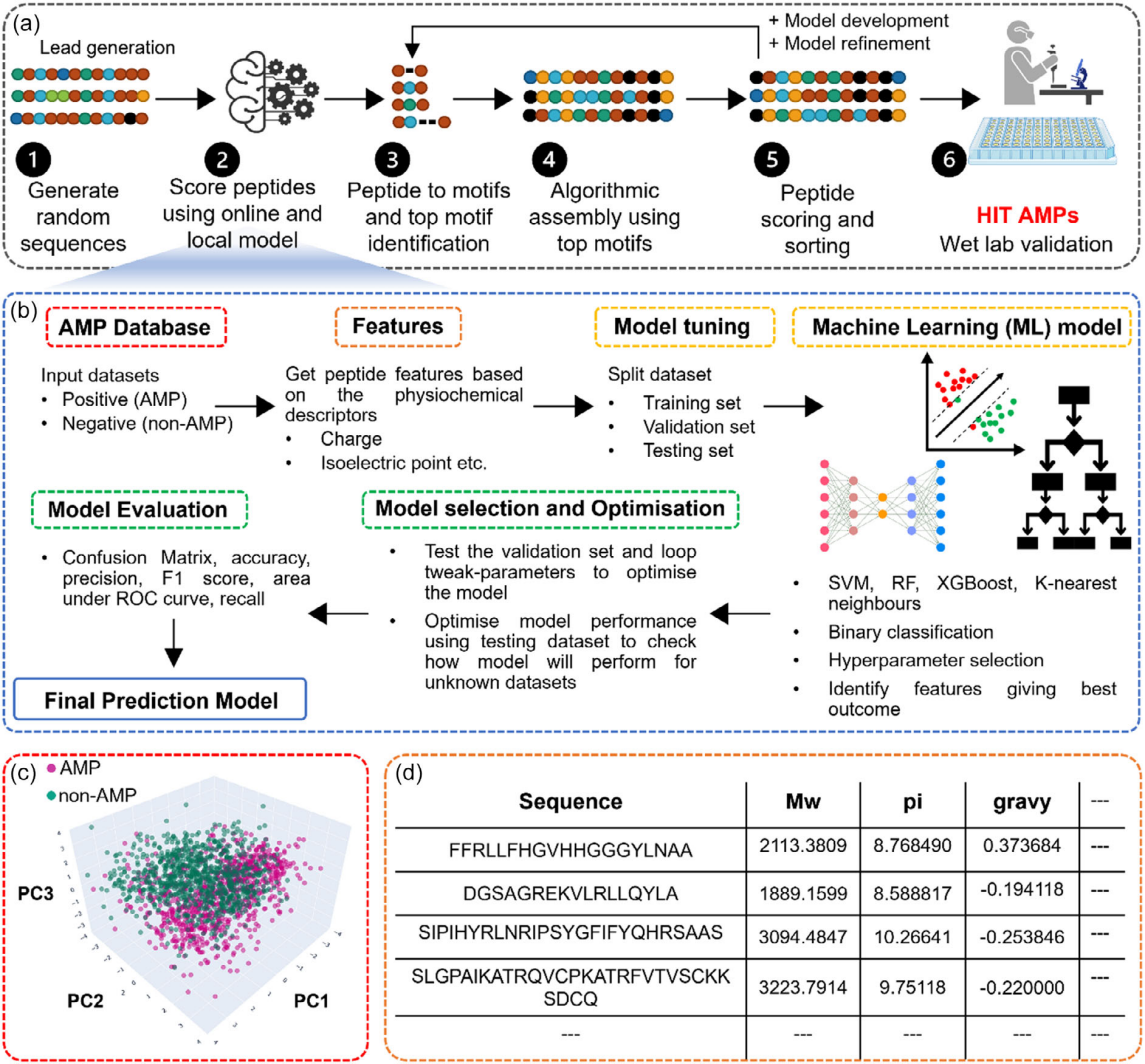
a) The workflow of algorithm depicting the generation of random peptides and scoring the peptides using ML models for prediction as AMP or non‐AMP, conversion of peptides to indicial motifs, identification of top motifs, and their assembling into new peptide sequences followed by experimental validation. b) Workflow explaining the training of ML models used for the prediction of antimicrobial activity. c) 3D PCA results of AMP and non‐AMP datasets. d) Example of physicochemical features used to describe the AMP and non‐AMP datasets in the form of numerical tabular‐based data.

As numerous prediction tools exist for AMP, we harnessed these existing online tools (iAMP and AntiBP2)^[^ ^27^ ^]^ alongside the development of our own prediction model. The online webservers utilized various peptide features as input to train SVM, quantitative matrix, and artificial neural network (ANN) model for classification and prediction.^[^ ^27^ ^]^ This combined model was used in tandem with a generative algorithm to create peptide sequences of various lengths, guided by the occurrence of specific motifs (**Figure** **1**a,b). The process is summarized in Figure 1a. In this instance, i) the program randomly generates peptide sequences of a chosen length and test this first generation against an in silico assay simulator or simulators which is our ML‐based prediction model (Figure 1b) in conjunction with the online prediction tools (iAMP and AntiBP2); ii) the simulator(s) assign a score based on similarity to an “ideal” sequence called the target sequence; iii) next, the program will generate peptide motifs of the specified length and, using the scores from the assay simulation, perform regression analysis to identify key important motifs; iv) then, key motifs are integrated into otherwise random sequences (or at later stages whole peptide sequences will be constructed from key motifs); and v) the new sequences are tested in the assay simulator using ML model and vi) validated experimentally.

For training and fine‐tuning, the local ML model for predicting and scoring the peptide sequences, we utilized 1968 known peptide sequences from existing databases (http://cabgrid.res.in:8080/amppred/about.html).^[^ ^8^, ^27^ ^]^ Figure 1b illustrates the workflow of training, validating, and testing of the various ML models in distinguishing AMP and non‐AMP.

The datasets consisted of 984 AMP and 984 non‐AMP (refer to method section for details).^[^ ^27^ ^]^ Figure 1c represents the PCA for visualizing the AMP and non‐AMP datasets. For the purpose of binary classification, AMPs were designated as 1 and non‐AMPs as 0. The combined peptide dataset was then subsequently divided into three separate sets: a training set, a validation set, and a testing set, each randomly selected from the AMP and non‐AMP datasets (Figure 1b).

For training the model, we utilized physiochemical and structural properties as peptides descriptors or features. Various features for all the known 1968 peptides used to train the ML model were calculated (Table S1, Supporting Information, and Figure 1d) using two python packages: BioPython (https://biopython.org/)^[^ ^28^ ^]^ and modLAMP (https://modlamp.org/).^[^ ^29^ ^]^ The Bio.SeqUtils.ProtParam module in Biopython and GlobalDescriptor/PeptideDescriptor modules in modLAMP provide a convenient way to extract information about a protein's composition, including its molecular weight, isoelectric point (pI), amino acid composition, and other physicochemical characteristics (such as aromaticity, charge, hydrophobicity, hydropathicity, etc.) (Table S1, Supporting Information).^[^ ^28^, ^29^ ^]^

**Figure 2:**
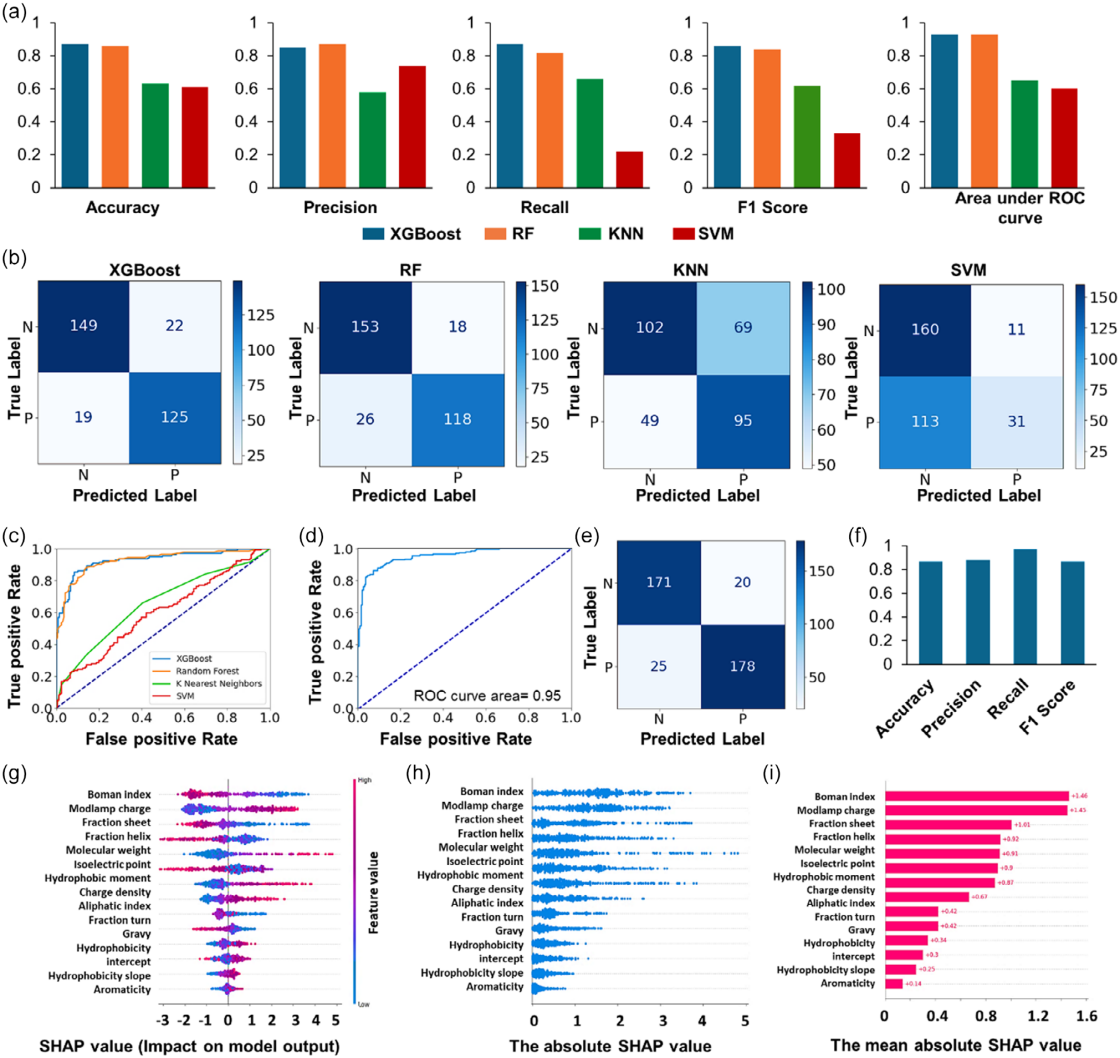
a–c) The validation dataset evaluation metrics showing the performance of various ML models: XGBoost, RF, KNNs, and SVM represented by (a) accuracy, precision, recall, F1 score, and area under ROC; (b) confusion matrices, where N and P are representing negative dataset as in non**‐**AMP and positive dataset as in AMP samples, respectively; and c) ROC curve. d–f) Test set performance metrics of XGBoost model represented by (d) ROC curve, (e) confusion matrix, (f) accuracy, F1, precision, and recall scores. g–i) SHAP values of the peptide features and their impact on the model using test dataset, where (g) SHAP values show how much each feature has contributed to the XGBoost model used for the prediction of AMPs, (h) the absolute, and (i) mean absolute SHAP values, respectively, of each individual feature.

Next, the eXtreme Gradient Boosting (XGBoost), RF, K‐nearest neighbors (KNN), and SVM model were each trained, and the model performance on the validation set was compared using the different evaluation metrics such as accuracy, precision, recall, F1 score (**Figure** **2**a), confusion metrices (where N and P are representing AMP and non‐AMP samples, Figure 2b), and a receiver operating characteristic (ROC) curve (Figure 2c). The high values for accuracy, precision, recall, F1 score, and area under the ROC curve (closer of 1) suggest that the classification model performs effectively in distinguishing between AMPs and non‐AMPs. The weighted harmonic mean of precision and recall, represented by the F1 score, signifies optimal performance when value is 1 and indicates poorer performance when value is 0. Figure 2a suggests that the highest weighted F1 score was achieved using XGBoost model.

Therefore, the XGBoost model was selected for prediction of AMP activity, after comparing the performance across four ML models on the validation dataset. It is a highly robust ML algorithm renowned for its superior predictive accuracy as demonstrated by evaluation metrics shown in Figure 2a–c. Since a model involves choosing the optimal hyperparameters which are used to learn the optimal parameters that correctly map the input features (independent variables) to the labels or targets (dependent variable), therefore, model parameters are estimated from data automatically, and model hyperparameters are set manually to help estimate model parameters. It provides feature importance scores and offers an extensive set of hyperparameters that can be adjusted to optimize model performance. Techniques such as grid search or random search can also be employed to discover the most effective hyperparameter combinations.^[^ ^30^ ^]^ The model performance can be improved through hyperparameter tuning using the validation datasets. In this case, the Tree‐Structured Parzen Estimator (TPE) algorithm was used to tune six XGBoost hyperparameters: the fraction of features to subsample (colsample_bytree), minimum split loss (gamma), L1 regularization (alpha), L2 regularization (lambda), maximum tree depth (max_depth), and minimum child weight (min_child_weight).^[^ ^31^ ^]^ After optimizing the model using the training dataset and defining it with the optimized parameters, its predictive ability in distinguishing peptide sequences as either AMPs or non‐AMPs using the testing dataset as unseen data was assessed.

The model's performance was assessed using the ROC curve on the testing dataset. The ROC area under the curve (AUC) was calculated to be 0.95 (Figure 2d), indicating excellent model performance across various classification thresholds. When evaluated against the testing dataset, comprising of 394 peptides (consisting of 191 non‐AMPs and 203 AMPs), the model correctly identified 178 peptides as AMPs and 171 as non‐AMPs, as illustrated in the confusion matrix, and there were only 20 false negatives and 25 false positives (Figure 2e). Overall, the model when evaluated using test dataset displayed an accuracy of ≈87%, with an F1 score of 0.8734 (Figure 2f). In this case, both precision and F1 score were ≈0.87, indicating a high degree of concordance between global accuracy and the weighted F1score.

Furthermore, the Shapley Additive Explanations (SHAP) values were used to quantify the individual contributions of each physicochemical feature to the model's outcomes. SHAP values originate from cooperative game theory and have been adapted for ML to provide insights into the outputs of intricate models.^[^ ^32^ ^]^ They elucidate how input features influence a model's predictions, that is, if they influence positively or negatively. Figure 1g offers insights into the impact of specific features, and notably, “overall charge”, “molecular weight”, and “charge density” were identified as factors with a positive correlation with the model's predictions. Conversely, “Boman index” (which is a measure for protein–protein interactions) “fraction sheet”, and “fraction helix” (associated with secondary structure) were among the features with a negative correlation with the model's predictions. Figure 1h,i provides an overview of the absolute and mean absolute SHAP values, both a useful measure of feature importance. This visualization highlights that “aromaticity”, “hydrophobicity slope and intercept”, as well as “gravy (grand average of hydropathy)” have minimal influence on the model's predictions.

**Figure 3:**
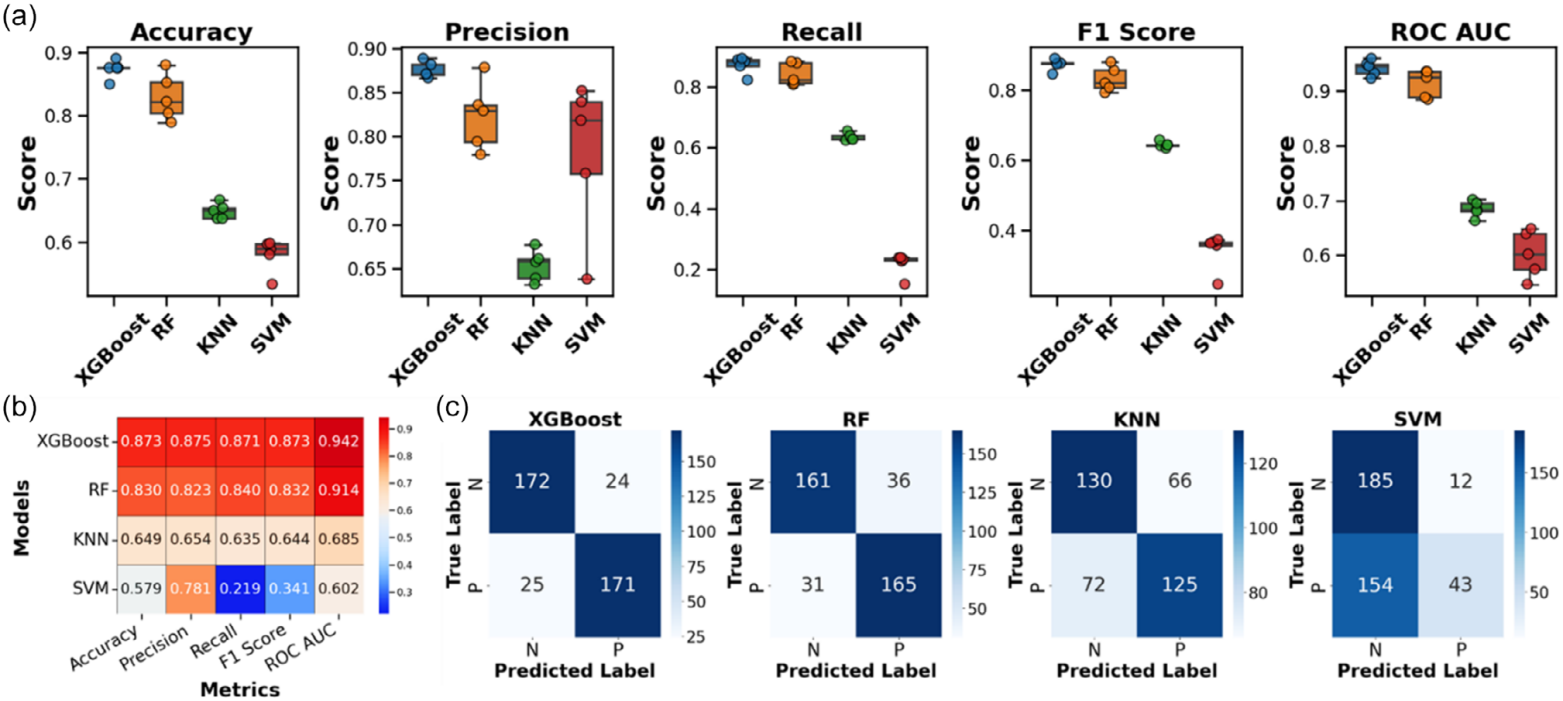
a) Box plots showing the values of various evaluation metrics accuracy, precision, recall, F1 score, and ROC AUC comparing the performance of XGBoost, RF, KNN, and SVM models across five cross‐validation folds. b) Heat map providing the comparison of various ML models using mean values of various metrics across five cross‐validation folds. c) Confusion matrices showing the average values across five cross‐validation folds for XGBoost, RF, KNN, and SVM models, where N and P are representing negative dataset as in non‐AMP and positive dataset as in AMP samples, respectively.

The hyperparameter selection and model performance were measured using nested cross‐validation.^[^ ^33^ ^]^ Nested cross‐validation follows the conventional cross‐validation technique with the addition that, for each fold of the dataset, the model's hyperparameters are independently optimized. This optimization process uses an additional cross‐validation loop, applied to the training set of each fold. The performance of each of the four models (XGBoost, RF, KNN, and SVM) was assessed using five‐fold nested cross‐validation on the AMP dataset. The average and standard deviation of the results across various evaluation metrics (accuracy, F1, precision, recall scores, ROC AUC) are shown in box plots in **Figure** **3**a and Table S2, Supporting Information. The confusion matrices for all five datasets for each of the four models are shown in Figure S1, Supporting Information. The mean of cross‐validation performance comparison of various models is shown in heatmap in Figure 3b and confusion matrices in Figure 3c. The mean performance metrics confirm the XGBoost model as the best choice, as it has the highest values across all metrics. The low standard deviation shows the model's parameters are stable to variations in the AMP dataset.

### 2.2 The in Silico Prediction and Generation of AMPs

**Figure 4:**
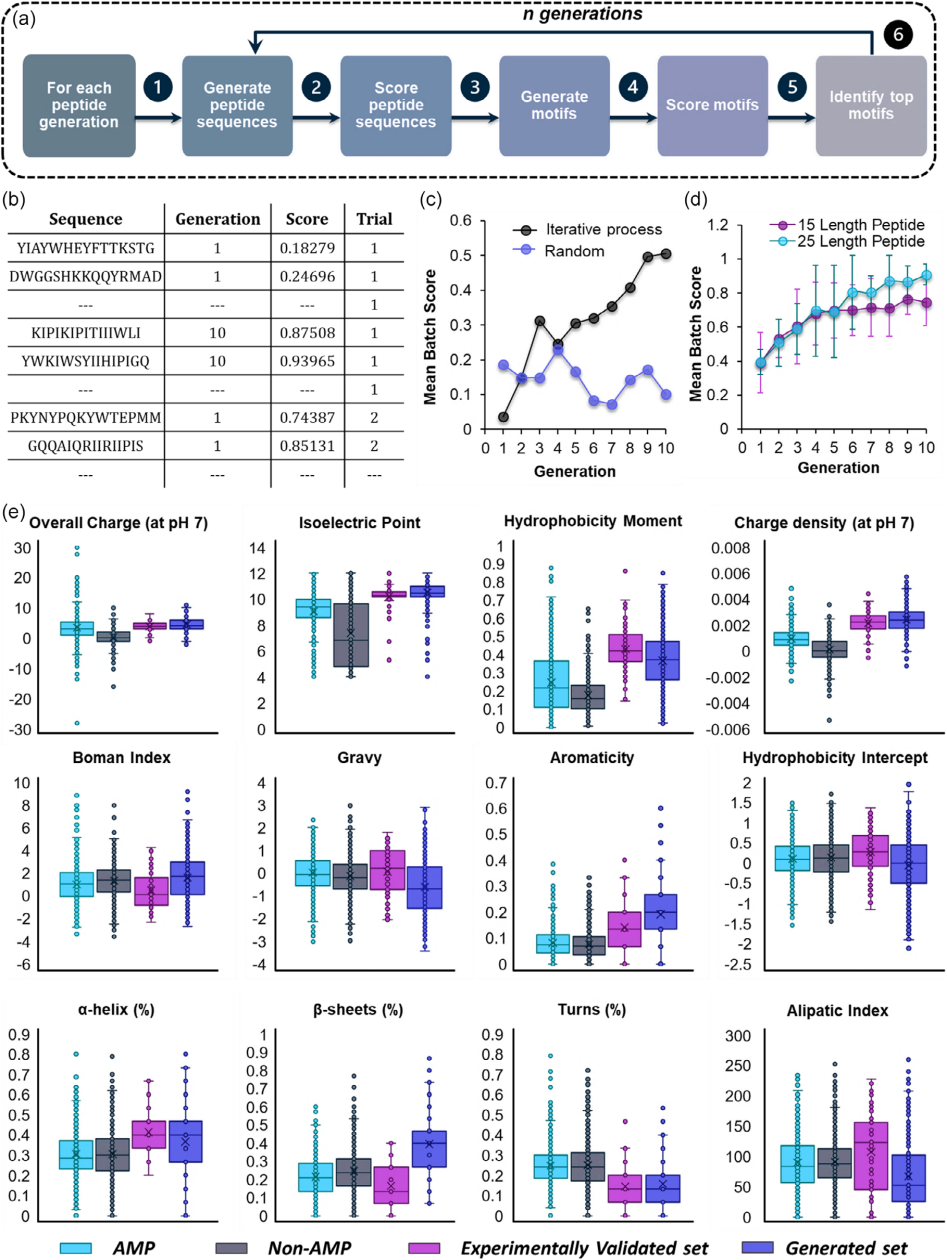
a) Scheme showing the sequence of algorithm for each generation of peptide set generated. b) Table showing the snapshot of the final output of the algorithm. c) Graph represents the mean batch score of peptide sequences generated either randomly or using iterative process algorithmic motif assembly. d) Mean score as a function of various generations across multiple trial tests of algorithm showing score reaching ≈1. e) The box plots shows comparison of distribution of AMP, non‐AMP, algorithmically generated, and experimentally validated peptide dataset across various physiochemical features.

Finally, once the prediction model for classifying peptides as AMP and non‐AMP was optimized, it was used in conjunction with an overall algorithm to generate new AMP sequences (Figure 1a). The process of generation of random peptides and scoring the peptides, conversion of peptides to indicial motifs, and identification of top motifs and then complying motifs into new peptide sequences was repeated with multiple selection, construction, and testing cycles to guide peptide discovery (**Figure** **4**a). The algorithm can be controlled by varying the number of peptides per batch, length of peptides, minimum and maximum motif, number of residues per motif, maximum number of alpha values and iterations to test in the lasso regression, and cutoff value for logistic regression weight for “important motifs”. The following sections discuss the overall algorithm and its output at each step in detail.

#### 2.2.1 Peptide and Motif Generation

Random peptides sequences, with both specific amino acid lengths (in this case 15) and batch number (5000), were generated using natural amino acids, namely “ACDEFGHIKLMNPQRSTVWY”. It is worth to mention that during peptide generation, we tried to exclude cysteine residues due to their synthetic challenges, multiple possible conformations, and association of disulphides with known toxic peptides like conotoxins.^[^ ^34^ ^]^

An illustration of this process, including the generation of random peptides and their scores (predicted antimicrobial activity) derived from the averaging of prediction scores obtained from online webservers and the local XGBoost model, is presented in Table S3, Supporting Information. The values closer to 1 signify high antimicrobial activity and closer to 0 mean low antimicrobial activity.

Following the peptide scoring process, the algorithm proceeds to the amino acid motif generation step. In the context of AMPs, motifs represent recurring amino acid sequence patterns or structural elements within a collection of AMP sequences. These motifs are often crucial for the antimicrobial activity of these peptides. To convert peptides into a set of motifs, each peptide is treated as a sequence of amino acids, to which Algorithm 1 shown in **Table** **1** is applied. The set of motifs can be tuned by four control parameters: the minimum and maximum allowable motif length and the minimum and maximum number of random amino acids to fill in empty space in the motif. These can be decided based on the initial length of the random peptide sequences. For this experiment, the minimum motif and maximum motif length were set at 2 and 10, and the minimum and maximum amino acids that would be used to fill up any empty spaces after making various attempts to merge various motifs were set to 2 and 4.

**Table 1:** Algorithm used for generating the motifs for peptides.

Algorithm 1: Motif generator		
in:	peptide $P=\langle a_{1},a_{2},\ldots ,a_{n}\rangle$ a sequence of *n* amino acids	
	minimum and maximum motif length $l_{min},l_{max}(0<l_{min}\leq l_{max}\leq n)$	
	minimum and maximum amino acids in motif $m_{min},m_{max}(0<m_{min}\leq m_{max}\leq l_{max})$	
	wildcard character, used to represent any amino acid *w*	
out:	a set of motifs *M*	
MOTIF‐GENERATOR$\left(P,l_{\min},l_{\max},m_{\min},m_{\max},w\right)$		
1.	M ← Ø	
2.	$\text{for }l\leftarrow l_{\min}\text{to }l_{\max}\text{ do}$	
3.	for all subsequences S of sliding window of width l over P do	
4.	$\text{ for }m\leftarrow m_{\min}\text{to }m_{\max}\text{ do}$	
5.	k ← max(l−m,0)	
6.	for all *k*‐combinations *K* of {1,2,…,l} do	
7.	$\;S^{\mathrm{new}}\leftarrow \text{copy }S$	
8.	for all i∈Kdo	
9.	$\;S_{i}^{new}\leftarrow w$	
10.	end for	
11.	$\;M\leftarrow M\cup \left\{S^{new}\right\}$	
12.	end for	
13.	end for	
14.	end for	
15.	end for	
Example		
	MOTIF‐GENERATOR(⟨A,D,E,F⟩,3,4,3,3,−)→	
	{⟨A,D,E⟩,⟨D,E,F⟩,⟨−,D,E,F⟩,⟨A,−,E,F⟩,⟨A,D,−,F⟩,⟨A,D,E,−⟩}	

#### 2.2.2 Motif Scoring and Ranking

Following the generation of the set of peptide sequences and set of motifs per peptide, each motif was then scored and ranked in accordance with their ability to best correlate with the high antimicrobial activity scores. For this purpose, the motif occurrences were calculated. To calculate the motif occurrence counts, the sets of motifs were generated for each peptide sequence, which were then all combined into a single set. For each motif, its occurrence as in number of times it occurred in each peptide was counted, resulting in single occurrence table for the whole peptide set (see **Table** **2**).

**Table 2:** Snapshot of output showing random peptides with motif occurrence columns and the peptide's respective predicted AMP score.

**Motifs**	**A‐‐‐‐‐‐‐‐A**	**A‐‐‐‐‐‐‐‐D**	**A‐‐‐‐‐‐‐‐E**	**A‐‐‐‐‐‐‐‐F**	**A‐‐‐‐‐‐‐‐I**	**A‐‐‐‐‐‐‐‐K**	**A‐‐‐‐‐‐‐‐L**	**A‐‐‐‐‐‐‐‐M**	**A‐‐‐‐‐‐‐‐N**	**A‐‐‐‐‐‐‐‐P**	…	**YYIH**	**YYP**	**YYP‐‐‐‐‐‐W**	**YYP‐‐‐‐‐A**	**YYP‐‐‐‐D**	**YYP‐‐‐R**	**YYP‐‐H**	**YYP‐P**	**YYPG**	**Score**
**Sequence**																					
**AEAETTTWIEGDQFQ**	0	1	1	0	0	0	0	0	0	0	…	0	0	0	0	0	0	0	0	0	0.06068
**AFEWMGHIIEERWPE**	0	0	1	0	0	0	0	0	0	0	…	0	0	0	0	0	0	0	0	0	0.24428
**AKNMPRWLYREPPMG**	0	0	0	0	0	0	0	0	0	0	…	0	0	0	0	0	0	0	0	0	0.09742
**AKPQAEVANLKSQVR**	0	0	0	0	0	0	1	0	0	0	…	0	0	0	0	0	0	0	0	0	0.00446
**AKYHEGNEPKVAKFE**	0	0	0	0	0	1	0	0	0	0	…	0	0	0	0	0	0	0	0	0	0.08715
…	…	…	…	…	…	…	…	…	…	…	…	…	…	…	…	…	…	…	…	…	…
**YKWAIGMQMTLFAPY**	1	0	0	0	0	0	0	0	0	0	…	0	0	0	0	0	0	0	0	0	0.62734
**YNPKYPIFHMMVFIL**	0	0	0	0	0	0	0	0	0	0	…	0	0	0	0	0	0	0	0	0	0.19927
**YRQRQIQPNFDHIYD**	0	0	0	0	0	0	0	0	0	0	…	0	0	0	0	0	0	0	0	0	0.57416
**YTTTHIIWNYGWQIA**	0	0	0	0	0	0	0	0	0	0	…	0	0	0	0	0	0	0	0	0	0.53008
**YVGGRGQEVRYSKMI**	0	0	0	0	0	0	0	0	0	0	…	0	0	0	0	0	0	0	0	0	0.18266

Although it is dependent on the set of peptide sequences it was generated from, occurrence table is usually sparse, where majority of sequences will be infrequent or not appear at all. Hence, an initial filtering process was applied to the table, to remove excess irrelevant motifs.

This motif filtering process consisted of three steps: 1) standardization of each motif count to a variance of one and 2) fitting a linear regressor with L1 penalty (aka lasso regression) to the generated peptide sequences, using the peptide's standardized motif counts as the independent variables, and the peptide's predicted antimicrobial likelihood score as the dependent. The L1 alpha parameter was optimized using a grid search with cross‐validation. 3) And finally, only the subset of motifs with a nonzero regression coefficient was retained.

In particular, to score the filtered motifs, a model was trained to predict the score column, and the assessment of motif identification and scoring was carried out using cross‐validation techniques. The peptide score was converted into binary classification labels (with a threshold set at 0.5). Then, logistic regression classifier models with elastic net regularization were employed in a *k*‐fold cross‐validation setup, where each experiment consisted of splitting the training dataset into *k*‐folds, with *k* being set to 5 in this study. In each iteration of this process, *k*‐1 folds were used to fit the model using stochastic gradient descent, while the remaining fold was held back for assessing the model's performance on the test set. Upon completing all *k* iterations, the model coefficients were averaged to obtain the mean coefficients across iterations. It is important to choose an appropriate value of *k*, ensuring that the test partitions are sufficiently large to be representative of the problem while allowing an adequate number of “train‐test” evaluations for a reliable estimate of the model's performance on unseen data. The motifs were then sorted by this average coefficient, and the largest motifs (i.e., the motifs that have a largest positive linear relation to AMP score) were selected.

Ultimately, this approach identified the top 100 motifs, which highlighted specific segments within the peptides that correlated with higher antimicrobial activity scores (Table S4, Supporting Information). The motif generations and ranking integrated into peptide generation process presented in this study offer several advantages over state‐of‐the‐art tools used like STREME and MERCI.^[^ ^35^ ^]^ For instance, MERCI has very strict pattern‐based approach based on motif frequency rather than predictive modeling for identifying conserved motifs, STREME is statistically rigorous algorithm used to generate motifs from proteins sequences, and it uses *P* values and motif enrichment for motif ranking. Our algorithm is best tailored for AMP motif discovery with customizable motif length and random amino acid substitution, along with motif scoring (correlated with antimicrobial activity) which ensures that they are aligned with functional relevance. The method incorporated the ML approaches L1‐regularized regression (lasso) and logistic regression with cross‐validation to rank and eliminate irrelevant motifs, improving interpretability which are not used in MERCI and STREME.

#### 2.2.3 Peptide Generation Using Top Motifs

The top 100 motifs were utilized for the generation of new peptide sequences. These peptide sequences were created by merging of various identified motifs, either without conflicts or after making *n* attempts to fill the blank spaces with random amino acids. The outcome of this process is the generation of a new set of peptides, which is detailed in Table S5, Supporting Information (snapshot of the output).

Finally, this entire process can be iterated multiple times, each iteration is referred to as generations (see Figure 4a). The summary of the resulting output is presented in Figure 4b, where peptides were generated with specific parameters, including a peptide length of 15, an initial batch size of 5000 random peptides, a batch size for peptide generation set at 25 (these are peptides generated from top motifs), a total of 10 generations, a minimum motif length of 2, a maximum motif length of 10, a range for filling in random amino acids between 2 and 4, and five attempts to incorporate motifs to sequence before randomly filling amino acids (trial represent the number of times algorithm is operated). Figure 4c illustrates a graph displaying the mean AMP prediction scores of batches of peptides generated either randomly or through the generative model. The results indicate that the iterative algorithm process significantly improved the quality of AMPs as the generations continued. Furthermore, Figure 4d showcases the batch mean scores averaged across 20 trials of the algorithm when operated to generate peptides of length 15 and 25. Notably, the algorithm successfully produced peptide sequences with AMP scores close to 1 with activity being increasing along the generation, highlighting its ability to generate new AMP sequences effectively. The advantage to this algorithm is that with guided selection, attainment of an effective peptide sequences proceeds much faster than could be achieved via random peptide generation (or phage display) alone. Additionally, the program generates a comprehensive report highlighting the key decision‐making steps in guiding the peptide selection. This allows for retrospective evaluation of proposed sequences and opportunities to investigate intermediaries. Other models like CAMPR3, AMP scanner, DeepAMP, and iAMPpred use RF, SVM, and CNN instead of gradient boosting.^[^ ^35^, ^36^ ^]^ They used physiochemical features for classification, in combination with ensemble learning techniques and deep learning techniques to learn complex sequence pattern. However, these models lack motif generation, interpretability, and generative design. The algorithm offers integration of XGBoost model for prediction, motif‐based ranking using ML‐based feature selection (to relate to antimicrobial activity), and motif‐informed AMP generation.

In this study, we further evaluated the key physicochemical characteristics of the generated peptides and experimentally validated peptides with AMP and non‐AMP dataset. We found that peptides showed low sequence similarity which was determined using sequence alignment using APD3 webserver with known AMPs (<50%) signifying the novelty of the sequences. However, peptide sequences bear the key physicochemical characteristics of the positive dataset, that is, cationic charge, high charge density, and hydrophobicity moment (Figure 4e). Figure S2, Supporting Information, shows the comparison of amino acids composition of top 100 generated and predicted peptides to the AMP dataset used to train the ML model.

### 2.3 Experimental Validation of Antimicrobial Activity of the Peptides

To validate the model experimentally, the algorithm was operated 40 times twice with above mentioned parameters, and two lists of peptide sequences were generated. The generated peptide lists were sorted in order of their AMP prediction score. The top 100 peptides (which include the top 50 peptides from each list) were selected for synthesis, and their antimicrobial activity was subsequently determined through experimental assays. The peptide sequences from both lists are provided in Table S6 and S7, Supporting Information. The ESKAPE pathogens, which are a group of pathogens known for their ability to escape the effects of antibiotics, were utilized for validating peptide sequences. The ESKAPE pathogens have been listed by the World Health Organisation (WHO) as priority pathogens in 2011 to combat AMR.^[^ ^25^ ^]^ They are responsible for a significant portion of healthcare‐associated infections and are a growing concern in the field of infectious disease control. Addressing the challenges posed by ESKAPE pathogens is crucial for public health, as these bacteria continue to evolve and develop resistance to multiple antibiotics, making it increasingly difficult to treat infections caused by them. Hence, peptide sequences which can potentially inhibit or kill any of the ESKAPE pathogens are potentially important in the fight against multidrug‐resistant bacteria.

**Figure 5:**
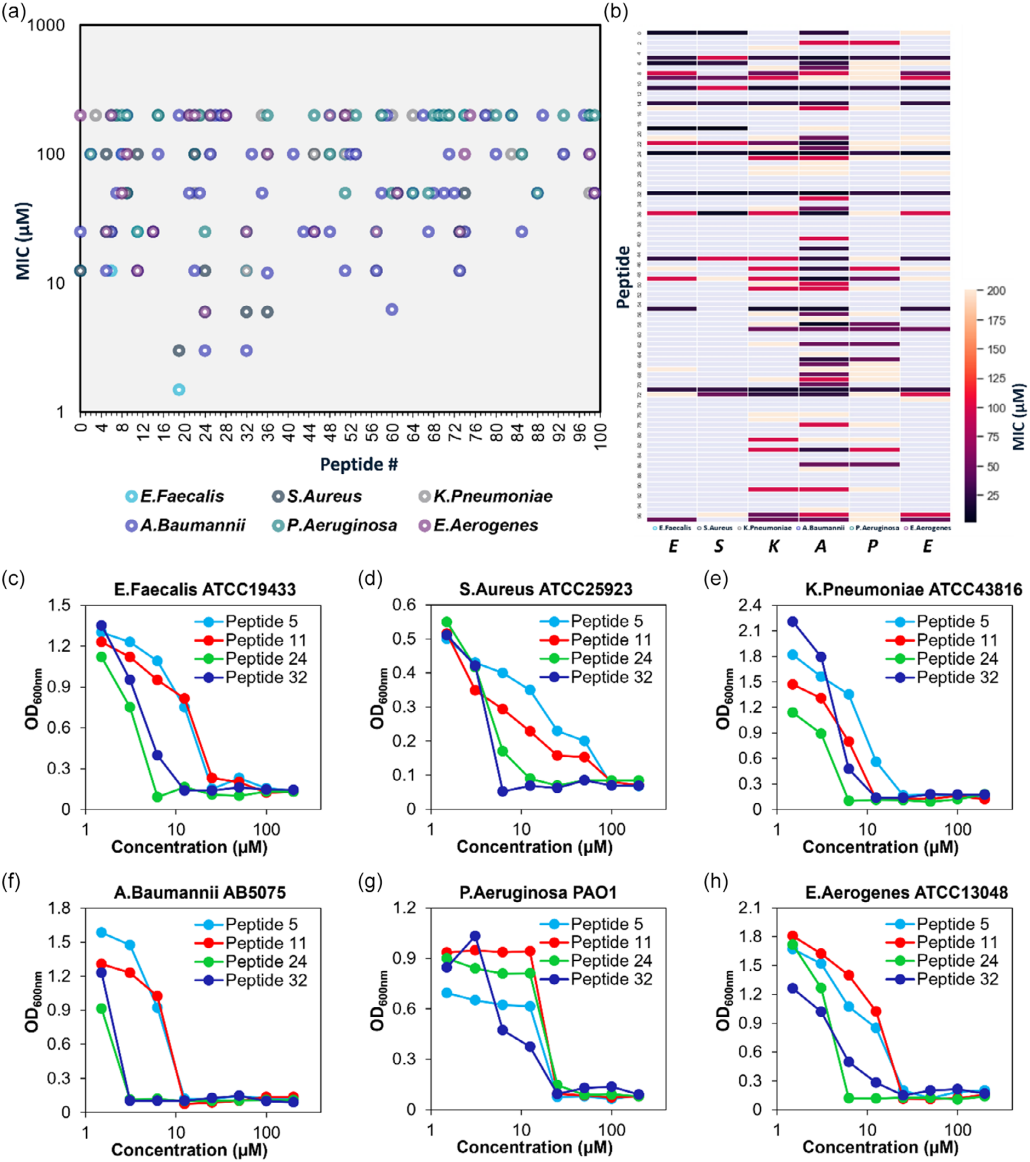
Antibacterial activity of the peptide generated using algorithm. a) The scatter plot of MIC values of various peptides against six different bacterial strains. b) The correlation plot of MIC values of various peptides against six different bacterial strains. c–h) Growth inhibition curves of ESKAPE pathogens represented as optical density (OD_600nm_) as function of concentration of peptides after 24 h incubation with (c) *Enterococcus faecium* ATCC19433, (d) *Staphylococcus aureus* ATCC25923, (e) *Klebsiella pneumoniae* ATCC43816, (f) *Acinetobacter baumannii* AB5075, (g) *Pseudomonas aeruginosa* PAO1, and (h) *Enterobacter aerogenes* ATCC13048.

To evaluate the susceptibility of the different peptide sequences against clinical isolates of various bacterial pathogens, we used the minimum inhibitory concentration (MIC) assay. The MIC measures the efficacy of peptides in inhibiting bacterial growth. The specific MIC values of various peptides against *Enterococcus faecium* ATCC19433, *Staphylococcus aureus* ATCC25923, *Klebsiella pneumoniae* ATCC43816, *Acinetobacter baumannii* AB5075, *Pseudomonas aeruginosa* PAO1, and *Enterobacter species* ATCC13048 were determined by measuring optical density at 600 nm (OD_600 nm_). **Figure** **5**a presents a scatter plot, and Figure 5b displays a correlation chart, illustrating that the MIC values of the peptides which were within the range of 1.5–200 μM. The percentage of peptides exhibiting activity against the specified bacterial strains were as follows: 23% against *Enterococcus faecium* ATCC19433, 17% against *Staphylococcus aureus* ATCC25923, 37% against *Klebsiella pneumoniae* ATCC43816, 55% against *Acinetobacter baumannii* AB5075, 37% against *Pseudomonas aeruginosa* PAO1, and 24% against *Enterobacter species* ATCC13048. Figure 5c–h shows representative growth inhibition curves of ESKAPE pathogens after incubation with some selected peptides (the peptides showing broad‐spectrum antimicrobial activity) at various concentrations as measured by OD_600 nm_. The growth inhibition curves were used to estimate the MIC values.

The overall accuracy of the model, considering the number of peptides active against at least one of the six bacterial strains, was estimated to be ≈60%.

**Figure 6:**
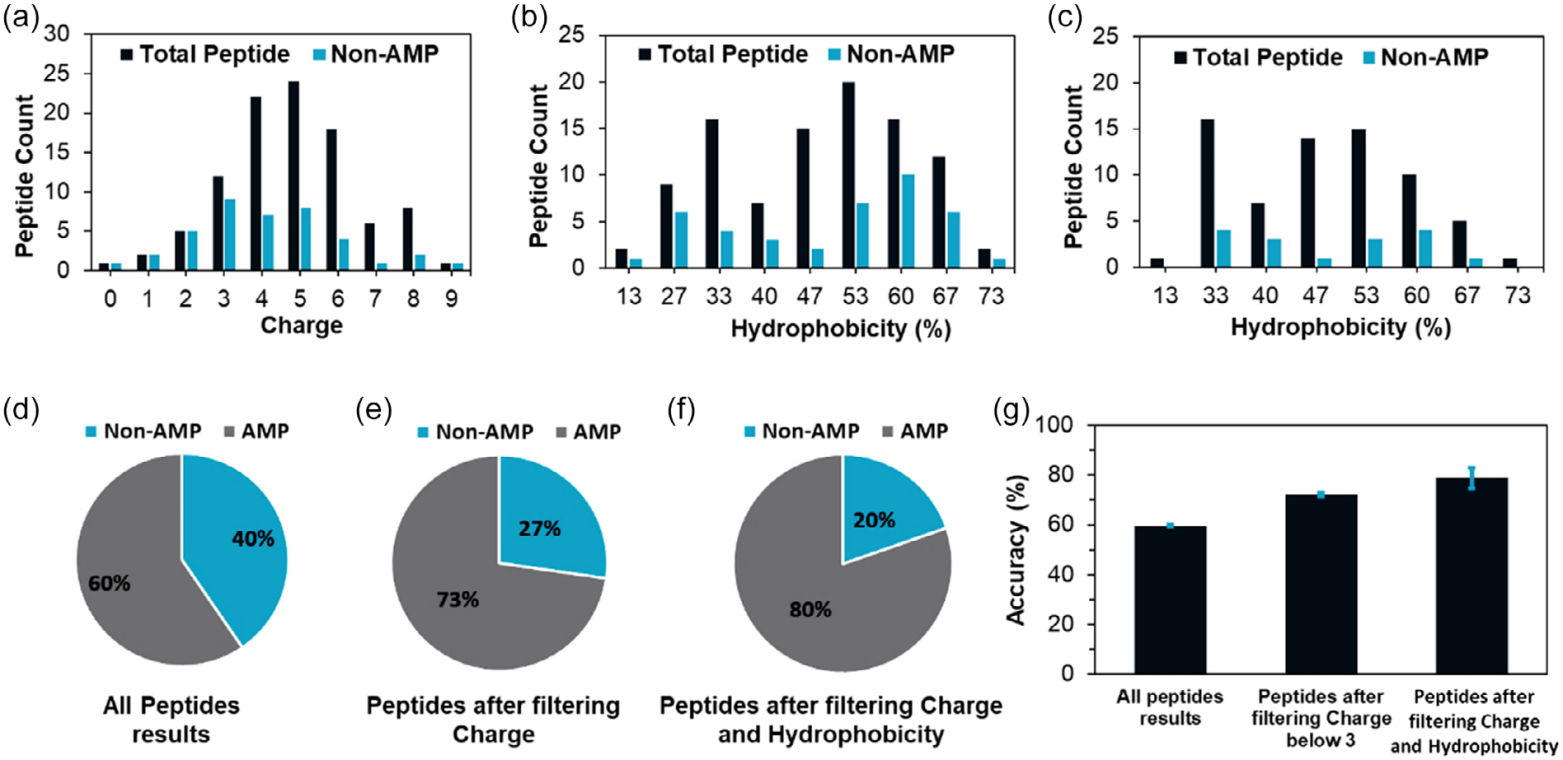
a) Charge distribution of all experimentally validated peptide sequences and only non‐AMPs. b) Hydrophobicity distribution of all experimentally validated peptide sequences and only non‐AMPs. c) Hydrophobicity distribution of all experimentally validated peptide sequences and non‐AMPs after filtering peptides with lower charge and hydrophobicity. d) Pie chart representing the percentage of AMP and non‐AMPs of experimentally validated peptide sequences. e) Pie chart representing the percentage of AMP and non‐AMPs after the applying the charge filter. f) Pie chart representing the percentage of AMP and non‐AMPs from experimentally validated peptide sequences after the applying both charge and hydrophobicity filter. g) The graph represents the accuracy of algorithm before and after using the filters based on physiochemical characteristics of experimentally validated peptide sequences.

Relative to the theoretical accuracy of the model, which was 87%, the experimental accuracy relative percentage was estimated to be ≈70%. During the testing phase, we conducted a comparative analysis of the physicochemical features of the experimentally validated peptides, with a particular focus on charge and hydrophobicity. The objective was to identify any discernible trends or patterns within the data with the scope to improve the algorithm, ultimately aiming to achieve higher accuracy. **Figure** **6**a,b illustrates the distribution of overall charge and hydrophobicity among two distinct sets of peptides. These distributions were compared with the number of non‐AMP peptides for relative analysis. Figure 6a reveals that a substantial proportion of peptides with charge values of 0, 1, 2, and 3 displayed limited effectiveness against the six bacterial strains. Likewise, most peptide sequences with a hydrophobicity value of 27% were found to be inactive. In response to these findings, additional filters were implemented to exclude data points falling within these specific charge and hydrophobicity from the dataset. Figure 6c illustrates the distribution of hydrophobicity after removing peptide sequences with lower hydrophobicity and charge showing that the number of inactive peptides was reduced.

Figure 6d presents accuracy percentages for both AMP and non‐AMP peptides across all the experimentally validated peptides. In comparison, Figure 6e,f demonstrates the enhanced percentage of AMP peptides achieved after applying the filters, specifically removing peptide sequences with charge values below +3 and hydrophobicity values of 27%. Figure 6g presents a bar graph showcasing the absolute accuracy percentages of the algorithm after applying the filters separately to two different sets of peptides. Remarkably, the minimal error bars indicate that implementing these filters across two different sets of peptide sequences led to a significant improvement in algorithm accuracy, from ≈60% to ≈80%. As a result, the algorithm was refined to incorporate all physiochemical features and apply a filter to remove peptides with lower hydrophobicity and specific charge values to enhance its accuracy. Our model after refinement provides better performance than other models in terms of accuracy and interpretability. Other models such as AMPscanner, CAMPR3 (RF), CAMPR3 (SVM), CAMPR3 (ANN) showed accuracy between 55% and 70% when analysis was performed on the experimentally validated dataset.[^35^, ^36^]

Furthermore, the top 50 peptides were also tested against the clinical isolate of various fungal strains, namely *Candida albicans* ATCC 90 028, *Cryptococcus neoformans* ATCC 208 821, and *Cryptococcus neoformans* ATCC 901 122, and ≈65% of the peptides showed activity in effectively inhibiting fungal growth (Figure S3a–c, Supporting Information). Next, the off‐target toxicity of the AMPs was determined using whole human blood using hemolysis assay and cell viability against human embryonic kidney cells HEK‐293 cells. The results suggested that only 6% of active peptides had cytotoxicity toward HEK‐293 cells and red blood cell hemolysis between concentration of 1.5 and 200 μM (Figure S4 and S5, Supporting Information). Moreover, to emphasize on the importance of peptide sequences and their amino acid residues generated using the algorithm, we tested scrambled sequences of the peptides and the scrambled peptide sequences did not show any antimicrobial activity. Overall, the generated algorithm offers a promising avenue for the rapid development and prediction of new AMPs to combat infectious diseases caused by antibiotic‐resistant microbial pathogens.

The study can be extended to further construct an algorithm to identify peptides active against specific bacterial strains. For instance, identifying patterns and the common motifs present in known peptide sequences targeting gram‐negative or gram‐positive bacterial strains would allow us to generate peptides with high specificity.

## 3 Conclusion

AMPs have emerged as promising candidates to be used against microbial pathogens and related infections due to their broad‐spectrum activity against various microorganisms. However, the experimental discovery and optimization of AMPs can be time‐consuming and expensive. In response to this challenge, current study utilized a combination of predictive ML models, cross‐validation, and top motif identification to build an algorithm for generating peptide sequences with high antimicrobial efficacy. The XGBoost model outperformed other models, RF, KNN, and SVM, and resulted in the highest accuracy of 87% in correctly predicting the peptide sequences as AMP and non‐AMP. The iterative process of motif ranking and assembly along with multiple selection, construction, and testing cycles used in the algorithm resulted in generating peptides with high theoretical scores. The wet‐lab validation of the top 100 generated peptides in inhibiting the growth of various bacterial and fungal strains which are prone to develop antimicrobial resistance showed the accuracy of 60%. Features like charge and hydrophobicity were considered to find trends for algorithm optimization, thereby improving predictive accuracy. Analysis revealed that peptides with low cationic charge and hydrophobicity exhibited limited antimicrobial activity. Filters were then applied to remove such peptides, leading to an enhancement in algorithm accuracy from ≈60% to ≈80%. Therefore, the generated algorithm offers a promising avenue for the rapid development of new antimicrobial agents to combat infectious diseases and antibiotic‐resistant pathogens.

## 4 Experimental Section

4.1

4.1.1

##### Materials

*N,N′*‐dimethylforamide (DMF) was purchased from VWR chemicals; acetic acid (AcOH), dichloromethane (DCM), formic acid (FA), isopropanol, ethanol, and HPLC grade acetonitrile (ACN) were purchased from Merck; diethyl ether (Et_2_O) was purchased from RCI Labscan; *N,N′*‐diisopropylcarbodiimide (DIC), piperidine, and trifluoroacetic acid (TFA) were purchased from Sigma‐Aldrich; RinkAmide AM resin (0.574 mmol g^−1^) was purchased from ChemImpex and Oxyma Pure; Fmoc‐protected amino acids/building blocks were purchased from Aapptec, Mimotopes, or ChemImpex. Bacterial pathogens *Enterococcus faecalis* ATCC19433, *Staphylococcus aureus* ATCC25923, *Klebsiella pneumoniae* ATCC43816, and *Enterobacter aerogenes* ATCC13048 were purchased from ATCC. Nutrient agar (OXOID, UK) and Mueller Hinton II Broth (MHB II, Cation‐Adjusted, BBLTM BD, France) were used for bacterial cultures in this study. HEK‐293 cells were purchased from ATCC. Fetal Bovine Serum (FBS) was purchased from Life Technologies, USA.

##### Data Collection and Preprocessing

The datasets consisted of 984 antibacterial (ABP) and 984 non‐antibacterial (non‐ABP) peptides. The datasets were taken from iAMPpred (iAMPpred: About (cabgrid.res.in). Particularly, peptides were collected from public database resources. Positive datasets from AntiBP2 (http://www.imtech.res.in/raghava/antibp2/), CAMPR3 (http://www.camp.bicnirrh.res.in/seqDb.php?page=0), and APD3 (http://aps.unmc.edu/AP/database/antiB.php) were used. Further, non‐antibacterial (negative dataset) was collected from AntiBP2 and AVPpred, respectively. The combined peptide dataset was then subsequently divided into three separate sets: a training set, a validation set, and a testing set, each randomly selected from the AMP and non‐AMP datasets. In addition, we also used a fivefold cross‐validation where datasets were randomly split into five groups containing equal number of AMPs and non‐AMP. The features such as aromaticity, gravy, molecular weight, isoelectric point, fraction helix, fraction turn, fraction sheet, hydrophobicity slope, hydrophobicity intercept, hydrophobic moment, Boman index, aliphatic index, charge density, and modlamp charge were used for model training. The two python packages, BioPython (https://biopython.org/) and modLAMP (https://modlamp.org/), were utilized for this purpose.

##### ML Models for Antimicrobial Activity Prediction

The XGBoost, RF, KNN, and SVM model were used, and they were trained using the above dataset, and the model performance on the validation set was compared. For XGBoost model hyperparameter optimization was performed using Bayesian optimization with the TPE algorithm (hyperopt package http://hyperopt.github.io/hyperopt/) using parameters like max_depth, min_child_weight, gamma, and colsample_bytree. For other models, hyperparameter tuning was performed using grid or random search techniques. Each model's performance was then evaluated on the testing dataset using the different evaluation metrics such as accuracy, precision, recall, F1 score, confusion metrices (shows the classification performance on AMP and non‐AMP dataset), and an ROC curve.

##### ML for Scoring Amino Acid Motifs

Motif occurrence counts to predict peptide antimicrobial activity score were utilized. An initial filtering process was applied. The ordinary logistic regression with fivefold cross‐validation was employed in identifying the most predictive motifs by taking the average value of the model coefficients across folds. The 100 top motifs were picked for the subsequent analysis and were related with the largest averaged coefficients. Sliding window regression and L1 regularization (decision rule) could simplify the model by erasing less predictive motifs. As a result, the analysis demonstrated that using a pipeline with StandardScaler and Lasso in the optimized GridSearchCV mode, significant motifs with non‐zero coefficients were present.

##### Code Implementation

Implementation of the code was performed using python 3.9.13 with different libraries such as scikit‐learn 1.1.3, modlamp 4.3.0, biopython 1.79, xgboost 1.7.1, hypyeropt 0.2.7, and shap 0.41.0.

##### Peptide Synthesis

Peptides were synthesized using SPPS Fmoc/tBu strategy with microwave synthesis conditions on a CEM Liberty Blue system (instrument uses stock solutions of all reagents) on a 0.1 mmol scale using five‐fold excess of Fmoc‐AA‐OH (0.2 m in DMF), 0.5 m DIC in DMF, and 1.0 m OxymaPure in DMF. Deprotection was performed with 20% piperidine and 0.1 m OxymaPure in DMF. For the final deprotection and cleavage from resin, the peptide resin was washed with DMF (3 × 1 min), DCM (3 × 1 min), and Et_2_O (3 × 1 min) and dried in a desiccator under vacuum. The cleavage cocktail was composed of TFA/H_2_O/TIS (95:2.5:2.5). 10 mL of cleavage solution was added to peptidyl resin, and the reaction was left for 1.5 h on an orbital shaker at 100 rpm. Resin was then filtered off, and peptides were precipitated with cold Et_2_O and centrifuged; crude peptide was then washed twice with cold Et_2_O and dried in a desiccator under vacuum for 30 min. Finally, crude product was dissolved in 10% AcOH and lyophilized before further analysis and purification. Peptides (1 mg mL^−1^) were analyzed on an analytical HPLC (with injection volume 10–20 μL) and purified on a preparative HPLC (with 5 mL injection volume containing ≈30 mg of peptide). The Agilent Technologies 1260 Infinity Series analytical HPLC was run at flow rate of 1 mL min^−1^, and wavelength of detector was set at 215/254 nm; column temperature was set to 30 °C. The column used was Phenomenex Luna 5 μm C18(2) 100 Å (100 × 4.6 mm) (PN, 00D‐4252‐E0; SN, 679 499‐7; BN, 5291‐122). Agilent Technologies 1260 Infinity was used for purification of crude peptide and was run at flow rate of 12 mL min^−1^ with detector wavelength set at 215/254 nm wavelength. The column used was Phenomenex Luna 10 μm C18(2) 100 Å (150 × 21.20 mm) (PN, 00 F‐4253‐P0‐ax; SN, 554 707‐1). The HPLC mobile phase was [A] 0.1% TFA in MQ water and [B] 0.1% TFA in ACN. Peptide samples were also analyzed using a Dionex 3000 HPLC system coupled to a Thermo Scientific Q Exactive mass spectrometer fitted with a HESI‐II ion source. Analyses were performed with a reversed phase HPLC column (ACE Excel 2 C18, 100 × 2.1 mm, 2 μm particle size), using an injection volume of 10 μl, a flow rate of 0.3 mL min^−1^, and a gradient (2% to 100% methanol in 15 min and then held at 100% methanol for a further 5 min). Both mobile phases contained 0.1% FA with column temperature of 40 °C.

##### MIC Assay

Bacteria: The MIC was the lowest concentration of a drug, which inhibited the visible growth of a bacteria and could be determined by reading the absorption at 600 nm (OD_600 nm_) using a spectrophotometer. The peptides were subjected to antibacterial testing against gram‐positive and gram‐negative pathogens. All the ESKAPE pathogens (*E. faecalis* ATCC19433, *S. aureus* ATCC25923, *K. pneumoniae* ATCC43816, *A. baumannii* AB5075, *P. aeruginosa* PAO1, and *E. aerogenes* ATCC13048) were inoculated from nutrient agar plates to obtain a bacterial suspension by incubating the colony of bacteria in a flask containing 10 mL of Mueller Hinton broth II (MHB) at 37 °C for 24 h with gentle shaking (75 rpm). The bacterial suspensions were then diluted in MHB to 1 × 10^6^ colony‐forming units (CFU) of bacteria mL^−1^. Next, using serial dilutions in 96‐well plates, different concentrations of each peptide from 1.5–400 μM were prepared. 100 μL of peptide solutions at final concentration ranging between 0.75–200 μM were then added to a 96‐well plate containing 100 μL of diluted bacterial suspension. After 18–24 h incubation at 37 °C, the turbidity of the samples was observed, and the optical density of bacteria was measured using OD_600 nm_. All assays were performed in duplicates. The MICs were calculated as ≥90% inhibition of bacteria growth compared to the positive control wells.

Fungi: *Candida albicans* and *Cryptococcus neoformans* were cultured on YPD agar at 35 °C for 24 and 48 h, respectively. A minimum of five single colonies were taken from each agar plate and dissolved in sterile water. The peptide solutions were serially diluted eight times in 384 deep‐well polypropylene plates (PerkinElmer; Cat. No. 6008690) and were transferred to test plates (384 well plates, PS, Corning; Cat. No. 3680). The solution of fungi was then adjusted to OD_530 nm_ = 0.3 and added to YNB media (2% glucose) to give a final cell density of 2.5 × 10^3^ CFU mL^−1^ in the test plates. The plates were covered and incubated at 35 °C for 36 h. MICs were determined by measuring optical density after 36 h incubation using the Biotek Synergy HTx Plate reader. *Candida albicans* plates were read at OD_630 nm_, and *Cryptococcus neoformans* plates were read at OD_600‐570 nm_ after addition of resazurin (final concentration 0.01%). The MICs were calculated as ≥80% inhibition of fungal growth compared to the positive control wells.

##### Red Blood Cell Hemolysis Assay

Whole blood (10 mL tube^−1^) was washed 2–3 times in 3 volumes of 0.9% NaCl (Baxter; AHF7124), with centrifugation for 10 min between washes, at 500 g, with reduced deceleration. A cell count was performed using a Neubauer hemocytometer, and then cells were diluted to 1 × 10^8^ mL^−1^ in 0.9% NaCl. Diluted cells (45 μL well^−1^) were added to the plates containing peptide solutions at various concentrations. Plates were sealed and then placed on a plate shaker for 10 min before being incubated for 1 h at 37 °C without shaking. After incubation, plates were centrifuged at 1000 g for 10 min, to pellet cells and debris, and then 25 μL of the supernatant was transferred into a 384‐well flat bottom polystyrene plate (Corning; Cat. No. 3680). Following transfer, absorbance (OD) was read at 405 nm using a Tecan M1000 Pro monochromator plate reader. Using nonlinear regression analysis of log (concentration) versus normalized hemolysis, using variable fitting, HC_50_ (concentration at 50% hemolysis) was calculated. In addition, the maximum percentage of hemolysis was reported. Primary human red blood cells are historically purchased by University of Queensland from Australian Red Cross. The use of human blood (sourced from the Australian Red Cross Blood Service) for hemolysis assays was approved by the University of Queensland Institutional Human Research Ethics Committee (approval number UQ 2020001239).

##### Cytotoxicity

HEK‐293 cells (45 μL) were cultured in 384‐well tissue‐culture‐treated plates at the seeding density of 5000 cells per well in Dulbecco's Modified Eagle Medium (DMEM) (Gibco; Cat. No. 11995073) supplemented with 10% FBS (GE; Cat. No. SH30084.03) and 100 U/mL each penicillin/streptomycin (Invitrogen; Cat. No. 15070063). The peptide solutions (5 μL) at the final concentration range of 200–1.56 μM were added to the cells, and the cell plates were incubated for 20 h at 37 °C, 5% CO_2_. After 20 h incubation, 5 μL of 100 μM resazurin (Sigma; Cat. No. R7017) in PBS was added to each well for a final concentration of 11 μM. The plates were then incubated for 3 h at 37 °C, 5% CO_2_. The fluorescence intensity was read using the TECAN Infinite M1000 PRO with excitation/emission 560/590 nm. Using nonlinear regression analysis of log (concentration) versus normalized cell viability, using variable fitting, IC_50_ (concentration at 50% cell viability) were calculated.

## Conflict of Interest

The authors declare no conflict of interest.

## Author Contributions

**Sukhvir Kaur Bhangu**: conceptualization: (equal); formal analysis: (lead); investigation: (lead); methodology: (lead); project administration: (lead); resources: (lead); software: (equal); supervision: (supporting); validation: (lead); visualization: (lead); writing—original draft: (lead); writing—review & editing: (lead). **Nicholas Welch**: conceptualization: (lead); data curation: (supporting); funding acquisition: (supporting); project administration: (supporting); supervision: (supporting); writing—review & editing: (supporting). **Morgan Lewis**: conceptualization: (supporting); methodology: (supporting); software: (supporting); validation: (supporting); visualization: (supporting); writing—original draft: (supporting); writing—review & editing: (supporting). **Fanyi Li**: methodology: (supporting); project administration: (supporting); validation: (supporting); writing—review & editing: (supporting). **Brint Gardner**: conceptualization: (supporting); investigation: (supporting); methodology: (supporting); project administration: (supporting); software: (supporting); supervision: (supporting); writing—review & editing: (supporting). **Helmut Thissen**: conceptualization: (supporting); formal analysis: (equal); funding acquisition: (lead); investigation: (supporting); methodology: (supporting); project administration: (equal); supervision: (supporting); writing—review & editing: (supporting). **Wioleta Kowalczyk**: conceptualization: (equal); formal analysis: (equal); funding acquisition: (equal); investigation: (supporting); methodology: (supporting); project administration: (supporting); resources: (supporting); supervision: (lead); writing—original draft: (supporting); writing—review & editing: (supporting).

## Supporting information

Supplementary Material

## Data Availability

The data that support the findings of this study are available from the corresponding author upon reasonable request.
