# Supplementary material for: Machine Learning‐Assisted Prediction and Generation of Antimicrobial Peptides
Source: Small Sci. 2025 Mar 6;5(6):2400579. doi: 10.1002/smsc.202400579 (PMC12168616; doi:10.1002/smsc.202400579)
Supplement: Supplementary file 1 — Supplementary Material [file SMSC-5-2400579-s001.pdf]

Supporting information

Machine learning assisted prediction and generation of antimicrobial peptides

Sukhvir Kaur Bhangu*, Nicholas Welch, Morgan Lewis, Fanyi Li, Brint Gardner, Helmut Thissen, Wioleta Kowalczyk*

S.K. Bhangu, N. Welch, F. Li, H. Thissen, W. Kowalczyk

CSIRO Manufacturing, Research Way, Clayton, Victoria 3168, Australia

E-mail: sukhvir.bhangu@unimelb.edu.au and wioleta.kowalczyk@csiro.au

M. Lewis

CSIRO Information Management & Technology, Kensington, Western Australia 6151, Australia

B. Gardner
CSIRO Information Management & Technology, Research Way, Clayton, Victoria 3168, Australia

Table S1: The physiochemical peptide features selected for model training.

Category	Property
Physiochemical	Overall charge (at pH 7)
	Charge density (at pH 7)
	Molecular weight
	Isoelectric point
	Aromaticity
	Gravy
	Boman index
	Aliphatic index
	Hydrophobicity profile – slope
	Hydrophobicity profile – intercept
	Hydrophobicity profile – moment
Secondary Structures	α-helix (%)
	β-sheets (%)
	Turns (%)

Table S2: The Table below shows the evaluation metrics (accuracy, precision, recall, F1, ROC AUC and confusion matrix's true and false negatives and positives) of four ML models across 5 Cross validation datasets and their respective mean and standard deviation.

XGBOOST	***1***	***2***	***3***	***4***	***5***	***mean***	***Std dev***
Accuracy	0.87564	0.87564	0.87564	0.84987	0.89059	0.87347	0.01314
Precision	0.87	0.86634	0.88144	0.87097	0.88833	0.87542	0.00818
Recall	0.88325	0.88833	0.86802	0.82234	0.89286	0.87096	0.02571
F1 score	0.87657	0.87719	0.87468	0.84595	0.89059	0.873	0.01466
ROC AUC	0.94906	0.93257	0.94553	0.92308	0.95944	0.94194	0.01276
True Negatives	171	170	174	172	175	172.4	1.85472
False Positives	26	27	23	24	22	24.4	1.85472
False Negatives	23	22	26	35	21	25.4	5.08331
True Positives	174	175	171	162	175	171.4	4.92341

Random Forest	***1***	***2***	***3***	***4***	***5***	***mean***	***Std dev***
Accuracy	0.85279	0.78934	0.82234	0.80407	0.88041	0.82979	0.033
Precision	0.83575	0.77941	0.82902	0.79412	0.87817	0.82329	0.03457
Recall	0.87817	0.80711	0.81218	0.82234	0.88265	0.84049	0.03299
F1 score	0.85644	0.79302	0.82051	0.80798	0.88041	0.83167	0.03215

ROC AUC	0.93561	0.88477	0.92489	0.88861	0.93544	0.91386	0.02256
True Negatives	163	152	164	154	173	161.2	7.57364
False Positives	34	45	33	42	24	35.6	7.39189
False Negatives	24	38	37	35	23	31.4	6.52993
True Positives	173	159	160	162	173	165.4	6.28013

KNN	***1***	***2***	***3***	***4***	***5***	***mean***	***Std dev***
Accuracy	0.64975	0.63706	0.63706	0.66667	0.65394	0.64889	0.01116
Precision	0.65775	0.63918	0.63235	0.67742	0.66129	0.6536	0.01615
Recall	0.62437	0.62944	0.65482	0.63959	0.62755	0.63516	0.01108
F1 score	0.64063	0.63427	0.64339	0.65796	0.64398	0.64405	0.00776
ROC AUC	0.6632	0.68379	0.68053	0.70178	0.69517	0.68489	0.01328
True Negatives	133	127	122	136	134	130.4	5.1614
False Positives	64	70	75	60	63	66.4	5.38888
False Negatives	74	73	68	71	73	71.8	2.13542
True Positives	123	124	129	126	123	125	2.28035

SVM	***1***	***2***	***3***	***4***	***5***	***mean***	***Std dev***
Accuracy	0.533	0.58883	0.59645	0.58015	0.59796	0.57928	0.02399
Precision	0.6383	0.81818	0.85185	0.75807	0.83929	0.78114	0.07835
Recall	0.15228	0.22843	0.2335	0.23858	0.2398	0.21852	0.03336
F1 score	0.2459	0.35714	0.36653	0.36293	0.37302	0.34111	0.04788
ROC AUC	0.54719	0.63929	0.6485	0.57472	0.60212	0.60236	0.03821
True Negatives	180	187	189	181	188	185	3.74166
False Positives	17	10	8	15	9	11.8	3.54401
False Negatives	167	152	151	150	149	153.8	6.67533
True Positives	30	45	46	47	47	43	6.54217

Table S3: Example of engine output for random peptide generation and their relative AMP score as determined using local and online prediction tools.

Sequence	Score
HFEIIDAMMVGKMEI	0.140870
ENHTYWDHIVDGDTR	0.889225
GVKQQSWDSDSQSVE	0.544579
WTTLNNSWRQGIIKY	0.532396
GWRYQHTFVHQLASM	0.844666
...	...
KPNDALDTWHMRETD	0.203518
PSMDWDEEGHTMFAP	0.051499
PYNLRTTMVVKGRKM	0.419810
IPASLYIHMQQVTFN	0.047382
WATMEPRWRAMEGIH	0.054740

Table S4: Snapshot of top 16 motifs identified using design engine.

```
'W----T',
'PY',
'M---M',
'L--A',
'Q-----V',
'Q--------S',
'FY',
'QQ',
'YM',
'M-Y',
'YQ',
'W-------T',
'WR',
'A-M',
'G---Q',
'W-------P',
```

Table S5: Snapshot of final list of peptides generated using top motifs with length 15 and batch size 25 with their respective AMP predicted score.

Sequence	Score
YFDYSNRWRYGILYR	0.082126
HYPDRALYVEFMVMW	0.072012
MPPWMLWIQWVVPYP	0.082951
QLMWQWVMSQKGWSV	0.141375
PLNNVFYVPYDDWMD	0.080597
VMMQYRMIVYGTAWG	0.550192
LGRMFWDDQYLFVHN	0.024660
RGLKTIDPYVYSHRT	0.876791
AWYHHFLWNHQYGPY	0.097113
WEKKRMVNQLTSMPN	0.050804
HAGWNWYVDTPTFKS	0.869051
VMWQWRETVYAQSNG	0.030749
GWTKQMQPGMTVQSF	0.936140
YQARWVQVELQVPFF	0.072117
LIWAVRLSHQTADIA	0.715828
HLQVSQAQIMWSHYI	0.128904
PMMFDQEQLMVVDVY	0.129583
WKPAKMWQWQNYVQP	0.670909
MLAVMPQSPVVDFSW	0.063223
WQDLENDARSNFKYT	0.182409
MTVQWEVQKSSDTMQ	0.026656
AYWRVRLTILMQWPY	0.092487
QKRVVQFQRSSPYPS	0.622008
QFRWWGLVSTPVPPW	0.758741

Table S6: List of peptides generated using algorithm and their respective antibacterial activities in terms of exact MIC value in µM.

Peptide name	Sequence	M.W.	Charge	*E. Faecalis ATCC19433*	*S. Aureus ATCC25923*	*K. Pneumoniae ATCC43816*	*A. Baumannii AB5075*	*P. Aeruginosa PAO1*	*E. Aerogenes ATCC13048*
MLWH01	LAKIVPHKIGKQLGT	1601.0111	4						
MLWH02	ALFLGGIWKKLPKAI	1653.0463	4				100	100	
MLWH03	GIKIIGIKVATFLKA	1570.0303	4			200			
MLWH04	LLQKIFVVKGKAGLK	1640.0833	5						
MLWH05	GLWKIKLAFGKMFAK	1736.0293	5	25	100	25	12.5	25	25
MLWH06	GKIFGKLFLALIKMW	1763.0652	4	12.5	25		25	200	200
MLWH07	GGAFFKLTQKIIKKA	1648.0158	5			200	50	200	
MLWH08	VQKLLKFKLKITAKA	1727.1515	6	100		50	100	200	50
MLWH09	LLGFTAFIKGQIKTA	1605.9577	3	50	50	100	200	200	100
MLWH10	GIVNVTGAITKLIKL	1537.9889	3						
MLWH11	AWLKFKKKFGLFAKI	1823.1305	6	25	100	12.5	12.5	25	12.5
MLWH 12	LVLQGIVKAVVKAIN	1563.0205	3						
MLWH 13	ILKLVANKIFHAVVF	1710.0677	3						
MLWH 14	WFKAIAIGLGKFIAK	1661.0151	4	25	25	25	25		25
MLWH 15	AVLIAKLFHKLNKIK	1734.1363	5	200		200	100	200	
MLWH 16	GGCCWRCNRRVLRCF	1826.8514	5						
MLWH 17	IAKVLNKFVFNGIIK	1702.0626	4						
MLWH 18	FGNLIATKQVKALNG	1571.9120	3						
MLWH 19	FLPLIILKFIGKLLP	1723.1493	3	1.5	3		200		
MLWH 20	LAFVKGILANVGKVK	1554.9944	4						
MLWH 21	IKVILKLVTNKGNGI	1608.0416	4	200		200	50		200
MLWH 22	TLVKAVAHIAKLLKF	1650.0677	4	100	100	50	12.5	200	200
MLWH 23	KFITKFVKGLGLGTL	1620.0096	4			200	50	200	
MLWH 24	TKFGQKVIKLIAQFL	1732.0731	4	6	12.5	6	3	25	6
MLWH 25	ALKAKLKFPGKFLFK	1734.1040	6			100	100	200	200
MLWH 26	GLLTTGKKKLLGIIA	1524.0096	4				200		
MLWH 27	VHKAFRKGSGAAILW	1638.9444	4			200	200		
MLWH 28	FVTITLKNFAKKKVG	1692.0419	5			200	200		200
MLWH 29	GIVKAQLNIATFLTK	1614.9791	3						
MLWH 30	KIVFGLAKIGKFIVK	1659.0931	5						
MLWH 31	FKLIKNVGALAFGTV	1575.9472	3						
MLWH 32	KGFAKMFRLLKKLAR	1805.1306	7	12.5	6	12.5	3	25	25
MLWH 33	NFKIIKKVGIKLKGA	1655.0942	6				100		
MLWH 34	LGAPLKWIKGKFAIP	1637.0151	4						
MLWH 35	KHVFAFLGGKLVKLK	1683.0681	5			200	50		
MLWH 36	AIVKKGWIKILAWVF	1770.1040	4	100	6	100	12	200	100
MLWH 37	LGKVAHWGKLHVPKL	1681.0275	4						
MLWH 38	FGLKPIKIGIKAIMI	1640.0543	4						
MLWH 39	FVLTKATNGVLKALI	1585.9889	3						
MLWH 40	KLKNKTAGIFKGQVK	1658.0325	6						
MLWH 41	YFHLKKSFGKKLKRV	1877.1483	7				100		
MLWH 42	FKRHGIGKFPKYASK	1762.0126	6						
MLWH 43	WFGKKFPQIACKRCC	1812.9075	5				25		
MLWH 44	GKFFAKALFLAIVKT	1652.0147	4						
MLWH 45	KLKGLIFAWFKGIIA	1703.0619	4	25	100	100	25	200	25
MLWH 46	IGIKGAMPLKGIIKK	1565.0184	5						
MLWH 48	GPLMGAFIKKLLPKF	1658.0075	4	200		100	25	100	200
MLWH 49	IAVNQLTKIAKVAVG	1522.9530	3						

Table S7: List 2 of peptides generated using algorithm and their respective antibacterial activities.

Peptide name	**Sequence**	**M.W.**	**Charge**	***E. Faecalis*** *ATCC19433*	***S. Aureus*** *ATCC25923*	***K. Pneumoniae*** *ATCC43816*	***A. Baumannii*** *AB5075*	***P. Aeruginosa*** *PAO1*	***E. Aerogenes*** *ATCC13048*
MLWH51	LGVKPVFKFFKFFGK	1787.062	5	100	200	100	12.5	50	200
MLWH52	KHFSGFNGFFKKFSK	1803.955	5			200	100		
MLWH53	LVLKIKKKAVKALWE	1765.167	5			100	100	200	
MLWH54	IIKIINVPNVIWNII	1760.104	2						
MLWH55	FIQMINIIIHYIHFI	1913.072	1						
MLWH56	GSKGSPFFSKKFRNF	1731.918	5						
MLWH57	QGWIKIILPHIRYIK	1876.153	4	25		12.5	12.5		25
MLWH58	AKKKVWAWTKWPPKI	1865.116	6			200	50	200	
MLWH59	KHGIPIYIKKITNKI	1764.111	5				200		
MLWH60	KTFIKKIGTKIKKWT	1818.157	7			200	6.25	50	
MLWH61	PSKYAKIAIKFHKQI	1770.064	5			50	50	50	50
MLWH62	KGGVWFGRILKHQSK	1739.008	5						
MLWH63	RFVKIGKGHGKKMMY	1777.993	6						
MLWH64	FKTLKHIKKFKRNPW	1969.186	7			200	50	50	
MLWH65	IQVGDIAYKKIKRIK	1771.116	5						
MLWH66	SKFAKNFKKKYKNKF	1904.112	8				200		
MLWH67	KIAKFVKKIIKPSIM	1742.133	6				25	50	
MLWH68	WVPVWPKKLKKLTRG	1834.143	6				50	200	
MLWH69	IVPIKKIIKIYKFQK	1857.23	6	200			200	200	
MLWH70	WKGKKKFFDKKKKWF	2027.195	8				50	200	
MLWH71	RQIDWYWKRKGKYAR	2052.125	6			200	100	200	
MLWH72	IMKKILINKIHNIMK	1835.133	5				50		
MLWH73	FNKGKKFFFKFIKWV	1962.136	6	25	25	12.5	12.5	25	25
MLWH74	RMFFKFKKKFKKWGQ	2032.168	8	200	50	25	25	200	100
MLWH75	GAKFFKDFKRKRDKV	1868.087	6						200
MLWH76	LHHIIIGIWKAIIYT	1789.074	2						
MLWH77	KKLHHPFFKHFSQKP	1904.066	5						
MLWH78	WKIVKKFMKSHSPYH	1914.042	5			200	200		
MLWH79	KKKFKITGHTKYDYG	1812.002	5			200			
MLWH80	FMKMVKKVMGKHKKF	1865.069	7				100	200	
MLWH81	IHAITHIIKKFDVIA	1717.037	2						
MLWH82	FSKIIGITLKGLTII	1615.04	3						
MLWH83	GFKKEFDKFWWKKKR	2056.149	6			100	200	200	
MLWH84	IIHLVIRWIGELATM	1763.025	1						
MLWH85	AKVWKKIIKKLKIKP	1819.262	8			100	25	100	
MLWH86	WIDIIEITIRIIVHI	1845.121	0						
MLWH87	PHFIKIIVQIIYIIN	1822.12	2						
MLWH88	KNRIHKHILKKIIYI	1915.233	6				50	50	
MLWH89	KFFKKKLKDKTKDWI	1951.174	6				200		
MLWH90	KGFGKYKKGKKIYKK	1799.127	9						
MLWH91	HPILKIIIIMDKHIY	1845.103	2						
MLWH92	FNKAGKFKNSKWNKI	1808.018	6						
MLWH93	NHTFQIFMRKGNKKY	1910.007	5			100	100	>200	
MLWH94	KQQKKFFKKKKFFKE	2015.216	8						
MLWH95	DKFVKKPWKKKVKKY	1948.21	8						
MLWH96	KAFKGFWKKGKHKDK	1831.07	7						
MLWH97	FKLPFNKFKKKSRKA	1865.148	8				200		
MLWH98	PKWHKNKMFKFKRKF	2048.174	8		200	50	100	200	100
MLWH99	VIYSIGLRIFNKIRP	1787.09	4	50		50	50	200	50
MLWH100	MHKVDHIIKKIMKTK	1848.092	5						

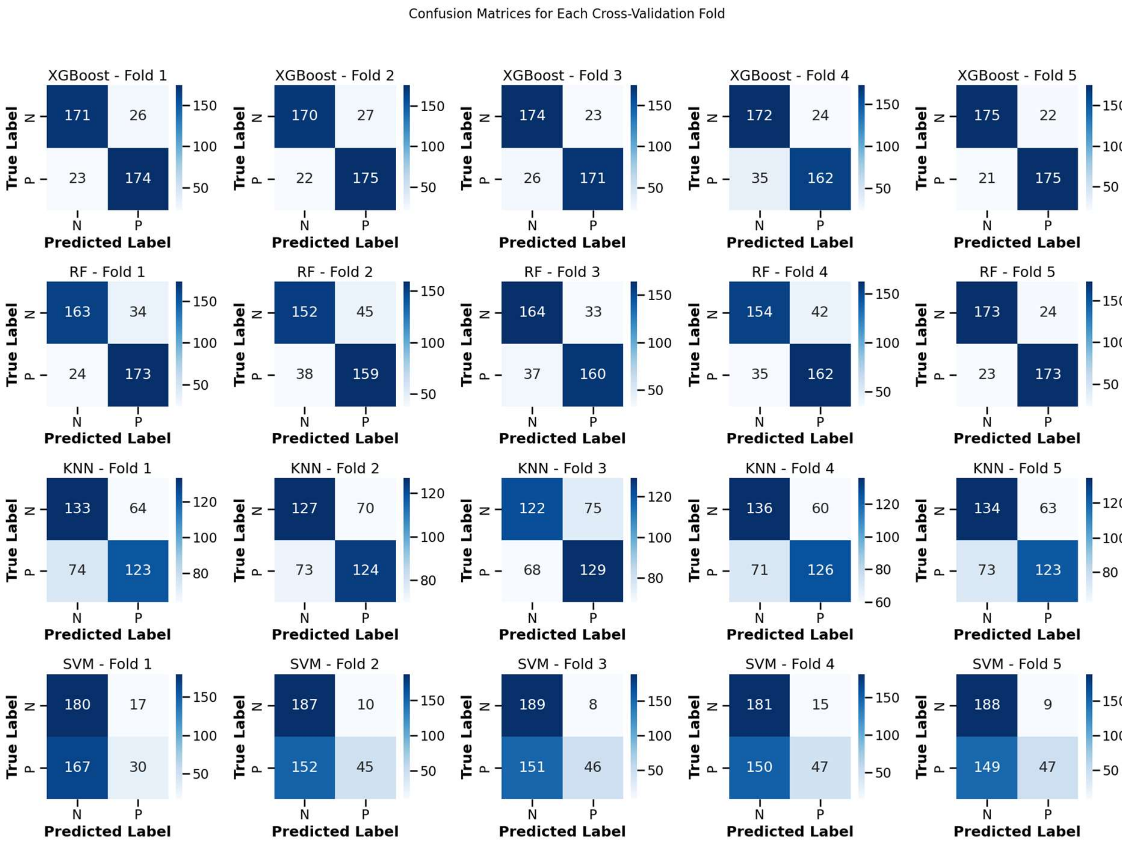

Figure S1: Confusion matrices for each Cross-Validation fold for four Models -XGBoost, RF, KNN, SVM

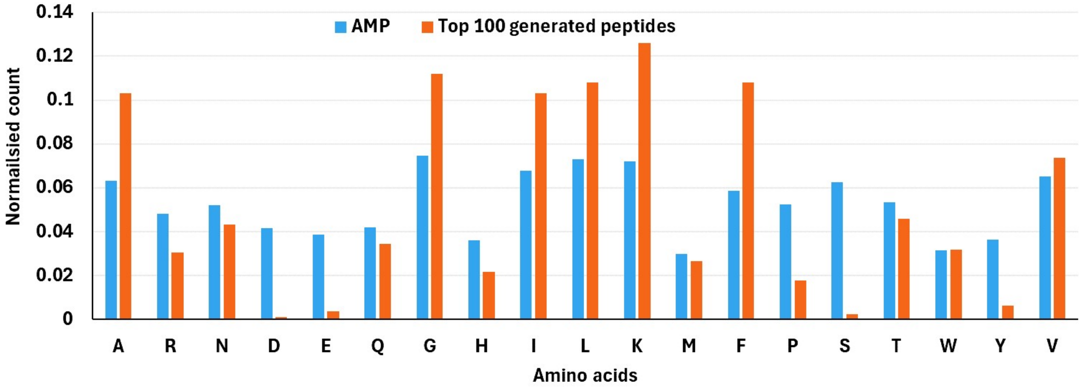

Figure S2: The comparison of amino acid composition of top 100 generated and predicted peptides to the training AMP dataset.

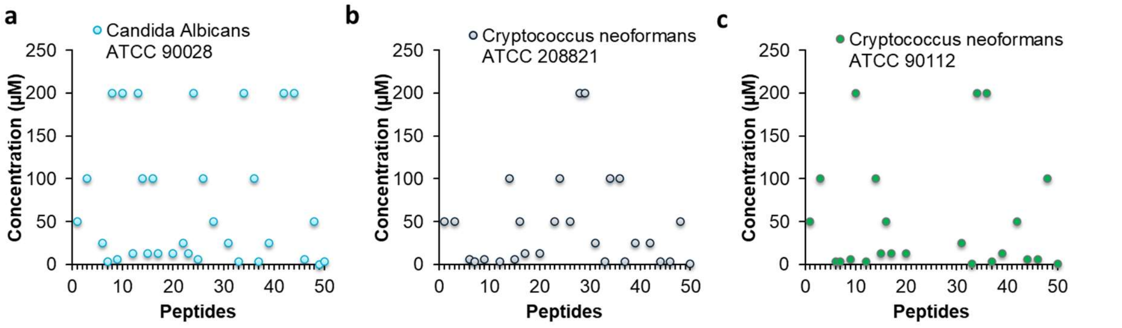

Figure S3: Antifungal activity of the peptide generated using algorithm. The scatter plot showing MIC values of various peptides against 3 different fungal strains, a) *Candida Albicans* ATCC90028, b) *Cryptococcus neoformans* ATCC208821*, and* c) *Cryptococcus neoformans* ATCC90112. The MIC testing experiments were performed in duplicates.

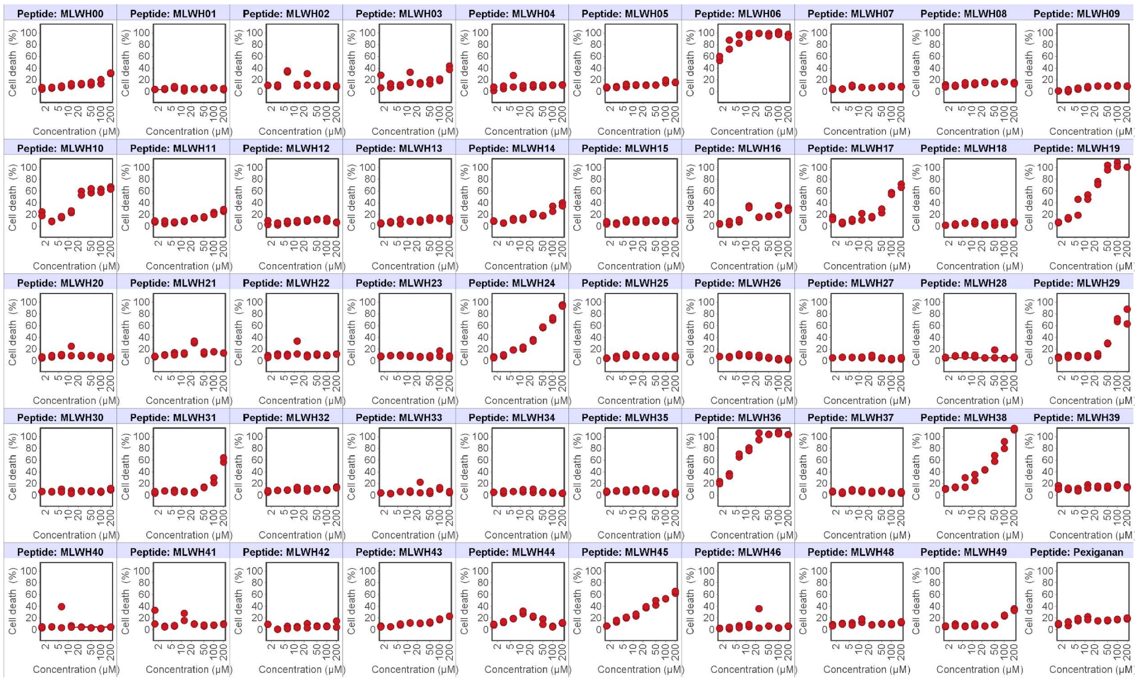

Figure S4: The graphs represent the hemolysis assay data for various peptides after incubation with whole human blood as a function of concentration. The experiment was performed in duplicates

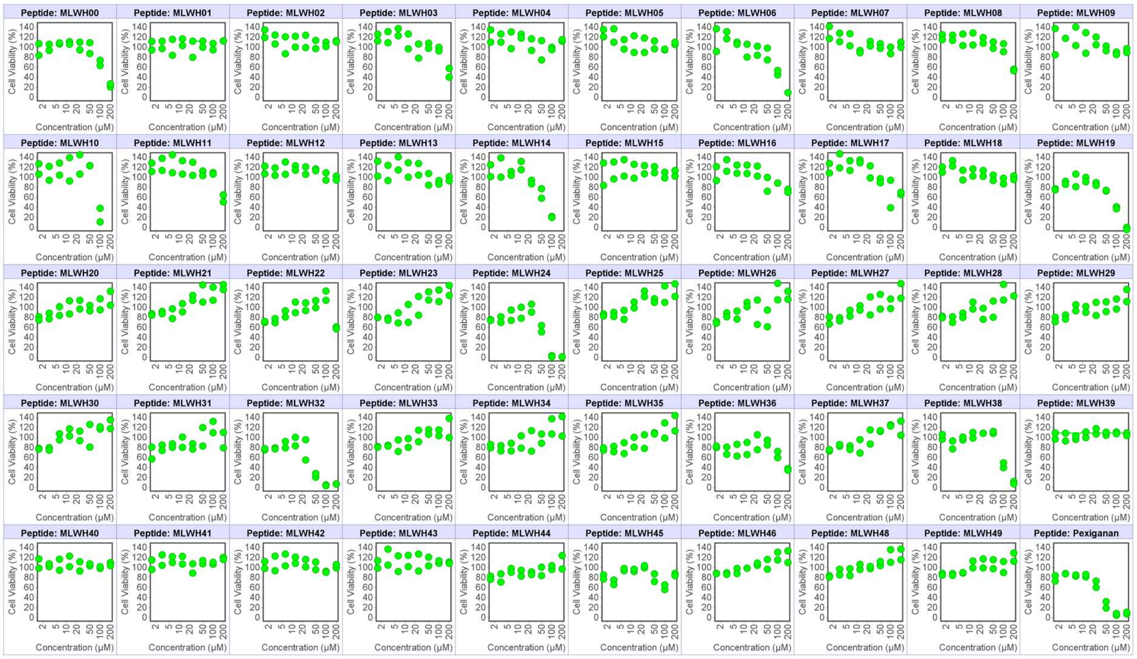

Figure S5: Cell viability of HEK-293 cells after 24 h incubation with various peptides as a function of concentration. The experiment was performed in duplicates
